# Comparison of the Performance of the TPTest, Tubex, Typhidot and Widal Immunodiagnostic Assays and Blood Cultures in Detecting Patients with Typhoid Fever in Bangladesh, Including Using a Bayesian Latent Class Modeling Approach

**DOI:** 10.1371/journal.pntd.0004558

**Published:** 2016-04-08

**Authors:** Kamrul Islam, Md. Abu Sayeed, Emran Hossen, Farhana Khanam, Richelle C. Charles, Jason Andrews, Edward T. Ryan, Firdausi Qadri

**Affiliations:** 1 Centre for Vaccine Sciences, International Centre For Diarrhoeal Disease Research, Bangladesh (icddr,b), Dhaka, Bangladesh; 2 Division of Infectious Diseases, Massachusetts General Hospital, Boston, Massachusetts, United States of America; 3 Department of Medicine, Harvard Medical School, Boston, Massachusetts, United States of America; 4 Division of Infectious Diseases and Geographic Medicine, Stanford University School of Medicine, Stanford, California, United States of America; 5 Department of Immunology and Infectious Diseases, Harvard School of Public Health, Boston, Massachusetts, United States of America; Oxford University Clinical Research Unit, VIET NAM

## Abstract

**Background:**

There is an urgent need for an improved diagnostic assay for typhoid fever. In this current study, we compared the recently developed TPTest (Typhoid and Paratyphoid Test) with the Widal test, blood culture, and two commonly used commercially available kits, Tubex and Typhidot.

**Methodology:**

For analysis, we categorized 92 Bangladeshi patients with suspected enteric fever into four groups: *S*. Typhi bacteremic patients (n = 28); patients with a fourfold change in Widal test from day 0 to convalescent period (n = 7); patients with Widal titer ≥1:320 (n = 13) at either acute or convalescent stage of disease; and patients suspected with enteric fever, but with a negative blood culture and Widal titer (n = 44). We also tested healthy endemic zone controls (n = 20) and Bangladeshi patients with other febrile illnesses (n = 15). Sample size was based on convenience to facilitate preliminary analysis.

**Principle findings:**

Of 28 *S*. Typhi bacteremic patients, 28 (100%), 21 (75%) and 18 (64%) patients were positive by TPTest, Tubex and Typhidot, respectively. In healthy endemic zone controls, the TPTest method was negative in all, whereas Tubex and Typhidot were positive in 3 (15%) and 5 (25%), respectively. We then estimated sensitivity and specificity of all diagnostic tests using Bayesian latent class modeling. The sensitivity of TPTest, Tubex and Typhidot were estimated at 96.0% (95% CI: 87.1%-99.8%), 60.2% (95% CI: 49.3%-71.2%), and 59.6% (95% CI: 50.1%-69.3%), respectively. Specificity was estimated at 96.6% (90.7%-99.2%) for TPTest, 89.9% (79.6%-96.8%) for Tubex, and 80.0% (67.7%-89.7%) for Typhidot.

**Conclusion:**

These results suggest that the TPTest is highly sensitive and specific in diagnosing individuals with typhoid fever in a typhoid endemic setting, outperforming currently available and commonly used alternatives.

## Author Summary

We compared the performance of the recently developed TPTest (Typhoid and Paratyphoid Test) with the Widal test, blood culture, and commercially available kits, Tubex and Typhidot in diagnosing the patients with typhoid fever. There is no acceptable gold standard; therefore, we estimated the sensitivity and specificity of these diagnostic methods with Bayesian latent class modeling. We found that the sensitivity and specificity of the TPTest is higher than other commonly used methods for diagnosis of typhoid fever.

## Introduction

Typhoid and paratyphoid fevers (collectively referred to as enteric fever) are caused by *Salmonella enterica* serovar Typhi (*S*. Typhi) and serovar Paratyphi A, B, C [1]. Enteric fever causes high morbidity and mortality worldwide [2,3]. The majority of cases of enteric fever are caused by *S*. Typhi, with approximately 22 million cases of typhoid fever occurring annually, resulting in over 100,000 deaths globally each year [2,4]. In endemic areas, the burden is highest in young children. The incidence of typhoid fever in slum residents in Dhaka, Bangladesh is approximately 2.0 episodes/1000 persons per year with a higher incidence found in children aged < 5 years of age (10.5/1000 person per year) than in older persons (0.9/1000 person per year) [5]. Clinical diagnosis of often enteric fever is difficult due to its non-specific nature (non-localizing febrile illness [6].

Unfortunately, accurate diagnosis of typhoid fever is problematic [7]. Several diagnostic approaches are commonly used, including microbiologic culturing of blood, and serologic assays such as the Widal or antigen-specific assays. All of these approaches suffer from poor sensitivity and/or poor specificity, especially in areas of the world endemic for enteric fever [7]. Many individuals with suspected typhoid fever are just empirically treated with antimicrobial agents, a clinical approach that drives development of microbial resistance, leaves individuals with other diagnoses without the correct treatment, and unnecessarily exposes patients to adverse effects of antibiotics. Although microbiologic culturing of bone marrow culture is considered a gold standard for diagnosing individuals with typhoid fever [8], it is clinically impractical due to its invasive nature [8]. As such, there is a pressing need for an accurate diagnostic assay for typhoid fever.

We have previously described development of an immunodiagnostic assay for enteric fever based on detection of anti-*Salmonella enterica* antibodies secreted by activated lymphocytes in the peripheral blood of acutely infected patients [9–11]. This assay, the TPTest (Typhoid and Paratyphoid Test), measures *S*. Typhi membrane preparation (MP)-specific IgA responses in peripheral blood mononuclear cell culture secretions [10,11]. Initial pilot analyses in Bangladesh have demonstrated high sensitivities (100%) and specificities (78%-97%), depending on the definition used, in identifying patients with enteric fever [10,11]. In this current study, we performed a direct comparison of the TPTest to blood culture, Widal analyses, and two common serologic assays, Tubex and Typhidot. We compared performance of the assays in 92 children and adults with suspected enteric fever in Dhaka Bangladesh, as well as in healthy endemic-zone controls, and a cohort of individuals febrile with non-typhoidal illnesses. Due to the absence of an acceptable gold standard, we used a Bayesian latent class modeling approach to estimate sensitivity and specificity of the various diagnostic approaches compared in this study [12,13].

## Materials and Methods

### Study participants and collection of blood

In total, we enrolled 127 participants in this study, including 92 participants who were clinically suspected of having enteric fever. Enrolment criteria for being a suspected enteric fever case included being 1–59 years of age, non-pregnant, having fever of ≥ 39°C for 3–7 days duration, and lacking an obvious alternative diagnosis. We collected 3–5 mL of venous blood for microbiologic culturing at the time of clinical presentation, and an additional 3 mL of blood for serologic analyses at clinical presentation and 7–28 days later. We also enrolled 20 healthy controls who also reside in Dhaka, Bangladesh, an area endemic for enteric fever, as well as 15 study participants who were febrile with non-typhoidal illness (visceral leishmaniasis and tuberculosis).

### Diagnosis of enteric fever by blood culture

We performed microbiological culturing of venous blood using a BacT/Alert automated system, sub-culturing positive bottles on MacConkey agar, blood agar, and chocolate agar plates, and identifying colonies using standard biochemical tests and agglutination test with *Salmonella*-specific antisera (Denka Seiken Co., LTD, Tokyo, Japan). Antimicrobial susceptibility testing of isolates was performed using the disc diffusion method following a modified Kirby-Bauer technique [14].

### Performance of immunodiagnostic assays

#### Widal agglutination test

We performed the Widal assay using 2-fold diluted plasma, reporting results as the reciprocal end agglutination titre. The Widal test assesses for the presence of antibodies reacting to the *Salmonella* flagellar (H) and/or lipopolysaccharide (O) antigens; we used a commercially available kit (Omega Diagnostics, Scotland,UK). We considered a positive result being a 4-fold increase in end titre from acute to convalescent phase of illness, or a single titre at either time point of ≥320.

#### Tubex TF and Typhidot assays

The Tubex TF (IDL Biotech AB, Karlsbodavägen 39, SE-168 11 Bromma, Sweden) assay is a serological test kit that detects IgM antibodies to *S*. Typhi O:9 lipopolysaccharide antigen [15]. The Tubex assay was performed for all participants following the manufacturer’s instructions, with scores of 4 or greater being considered as positive. The Typhidot assay (Reszon Diagnostics International Sdn. Bhd. Malaysia) is a dot-Enzyme Immunoassay (EIA) that assesses for the presence of IgM and IgG antibodies responses to a specific 50 kDa outer membrane protein (OMP) antigen of *S*. Typhi [16,17]. The assay was also performed following manufacturer’s instructions.

#### TPTest

The TPTest (Typhoid and Paratyphoid test) was carried out with blood samples from all participants; samples were collected in a sodium heparin tube as previously described [9–11]. Peripheral blood mononuclear cells (PBMCs) were separated by density gradient centrifugation on Ficoll-Isopaque (Pharmacia, Uppsala, Sweden) [10,18]. Isolated PBMCs were cultured at 10^7^cells/mL in RPMI complete medium [RPMI 1640 (Gibco, Gaithersburg, MD)] at 37°C and 5% CO_2_. After 48 hours, culture supernatants were collected and tested for IgA antibodies specific to *S*. Typhi MP by an enzyme-linked immunosorbent assay (ELISA) method as previously described [9–11]. We read the plates kinetically at 450 nm for five minutes at 19-second intervals, and expressed the maximal rate of optical density (OD) change as milli-optical density absorbance units per minute (mAB/min). We used pooled convalescent plasma from previous patients with known typhoid fever as a positive control on each plate to correct for variations between plates and different days of testing, and divided kinetic reading by this pool, expressing results as ELISA units as previously described [9–11]. Results >10 ELISA Units (EU) were considered positive, using a previously established cut-off value derived from a geometric mean plus two standard deviations of healthy Bangladeshi controls [9–11].

### Statistical analysis

We calculated the sensitivity and specificity with 95% confidence interval of the diagnostic methods using OpenEpi version 3.

### Latent class modeling

We then estimated the sensitivity and specificity of each of the diagnostics using a Bayesian framework with latent class models. For prior information, we assumed that the sensitivity of culture was 40–80% (95% confidence interval) and specificity was 100% [7].We used prior estimates of sensitivity of Tubex and Typhidot from a recent meta-analysis; mean (69%) and 95% confidence interval (45–85%) were estimated for Tubex in that analysis, but not Typhidot [19]. We therefore assumed that a range of previously established estimates (56–84%) reflected the 95% confidence interval for Typhidot. For prior estimates on specificity among all tests except culture, we used data from healthy controls and individuals with fever and confirmed alternative etiologies. We used a Gibbs sampler to sample from conditional parameter distributions using 100,000 Monte Carlo iterations; we discarded the first 50,000, and used the remainder for inference. Multiple chains were run and results examined to ensure convergence.

### Ethics statement

This study was approved by the research review and the ethical review committees of the icddr,b, and Institutional Review Board of the Massachusetts General Hospital. Written informed consent was obtained from all adult participants ≥18–59 years of age, and from parents or guardians of children 1–17 years of age.

## Results

### Characteristics of study participants

Of the 92 study participants with suspected enteric fever, 48 (52%) were male (Table 1). The median age was 6 years and 3 months, with a range of 1 to 46 years. The median temperature at enrollment was 39.2°C. The mean duration of fever before enrollment was 4 days. Reported symptoms and signs included headache (79%), abdominal pain (55%), constipation (30), coated tongue (51%), diarrhea (13%), vomiting (9%), non-specific rash (9%), and rose spot (7%).

**Table 1:** Characteristics of the patients with signs and symptoms of typhoid fever (n = 92).

Characteristics	Values
Median age in year (25th, 75th centile)	6.3 (3.3, 12.8)
No. of males (%)	48 (52.2%)
Median temperature in °C (25th, 75th centile)	39.2 (39.1, 39.5)
Duration of fever in days at presentation (25th, 75th centile)	4 (3, 5)
Patients with headache	79%
Patients with abdominal pain	55%
Patients with constipation	30%
Patients with coated tongue	51%
Patients with diarrhea	13%
Patients with vomiting	9%
Patients with rose spot	7%
Patients with rash	9%

### Blood culture and antibiogram

*S*. Typhi was isolated from the peripheral blood of 28 (30%) of the 92 suspected enteric fever cases patients; of these culture positive patients11 were male (39%) and 17 were female (61%). Resistance to antimicrobial agents was common. Of the 28 *S*. Typhi isolates resistance to ampicillin, trimethoprim-sulfamethoxazole, chloramphenicol, ciprofloxacin, and nalidixic acid was 15 (54%), 15 (54%), 17 (61%), 9 (32%), and 27 (93%), respectively. All isolates remained susceptible to cefixime, ceftriaxone and gentamicin. No *S*. Paratyphi were isolated by blood culture during the course of this study.

### Widal test

Among the 92 study participants with suspected enteric fever, seven were positive for a 4-fold change of Widal titre from acute to convalescent phase of illness, and 13 patients had a titre of ≥ 1:320. None of the patients with a 4-fold change had a titre ≥ 1:320, and no one with a positive Widal (defined as either a 4-fold change or single titer ≥ 1:320) had a positive blood culture.

### Grouping of the study participants

Based on the results of blood culture and Widal testing, we divided our 92 study participants with suspected enteric fever into four groups for further analysis: Group I- patients with a positive blood culture for *S*. Typhi (n = 28); Group II- patients with a fourfold change of Widal titre (n = 7); Group III- patients with Widal titre ≥1:320 (n = 13); and Group IV- suspected patients with a negative blood culture and a negative Widal test (n = 44). Interestingly, none of the 92 patients met criteria for more than one cohort. For comparison, we also created a Group V- healthy endemic zone controls (n = 20), and a Group VI- patients with other febrile illnesses (visceral leishmaniasis and tuberculosis) (n = 15).

### Comparison of TPTest, Tubex and Typhidot assays

**Fig 1:**
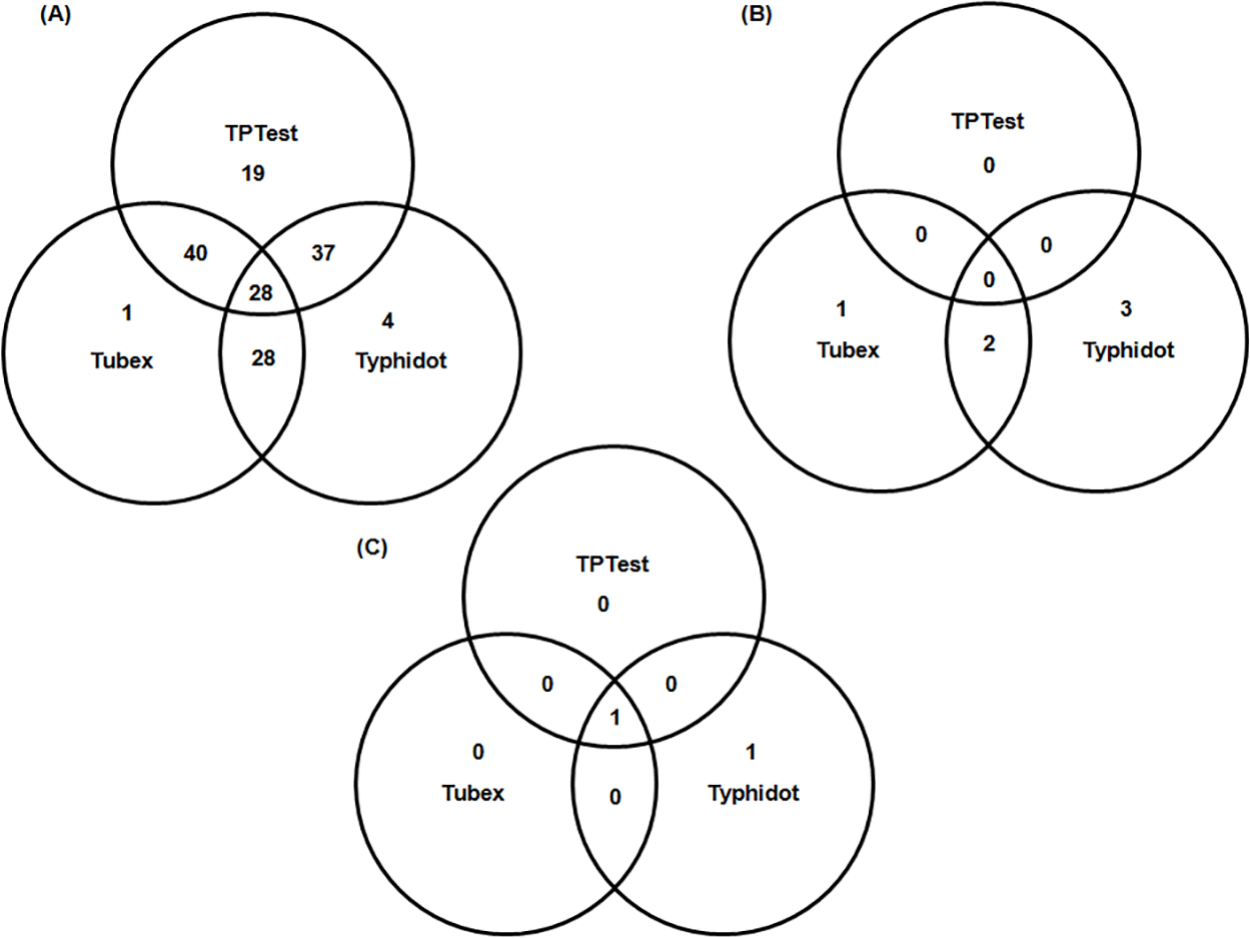
Venn diagram indicating (A) the patients in the six groups, (B) the healthy controls and (C) the patients with other febrile diseases are positive with each of the three tests (TPTest, Tubex and Typhidot).

**Fig 2:**
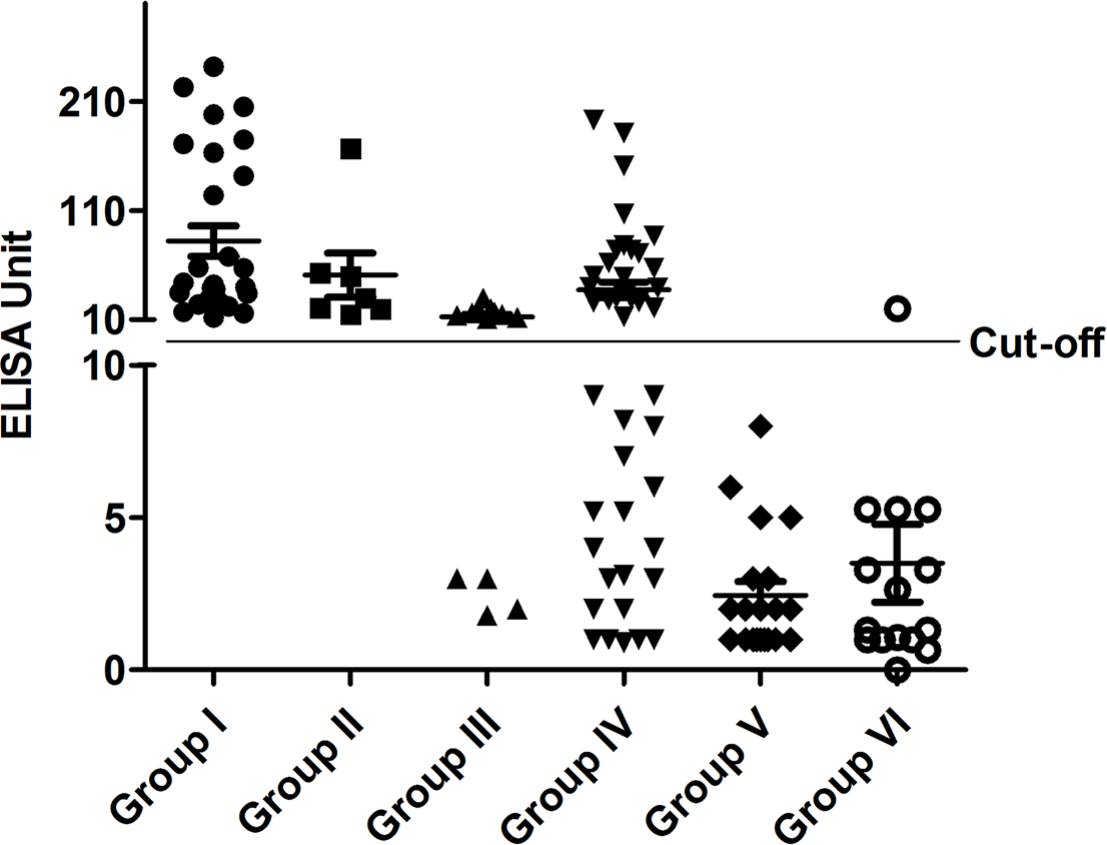
TPTest data point in different groups of study participants. The TPTest was carried out in different groups of the participants. The cut-off value of the TPTest was 10 ELISA unit. Groups: Group I, blood culture positive; Group II, four-fold change of Widal titre; Group III, Widal titer of ≥320; Group IV, blood culture and Widal test negative; Group V, healthy endemic zone control; Group VI, other febrile disease.

Out of 28 *S*. Typhi bacteremic patients, 28 (100%), 21 (75%) and 18 (64%) patients were positive by TPTest, Tubex and Typhidot, respectively (Table 2; Fig 1; Fig 2). For the four-fold change of Widal titre, the TPTest, Tubex and Typhidot were positive in 7 (100%), 6 (86%), 5 (71%), respectively. In case of patients with Widal titre ≥1:320, the TPTest, Tubex and Typhidot were positive in 9 (69%), 5 (38%), 3 (23%), respectively. The TPTest method was negative for all healthy controls, whereas Tubex and Typhidot were positive in 3 (15%) and 5 (25%), respectively. When considering patients with other febrile illnesses, the TPTest, Tubex and Typhidot were negative in 14 (93.3%), 14 (93.3%), and 13 (86.7%) patients, respectively. Among 44 participants who were negative by blood culture and Widal test, but clinically diagnosed as enteric fever, 24 (55%), 9 (20%), and 15 (34%) were positive by TPTest, Tubex and Typhidot, respectively.

**Table 2:** Comparison of results among TPTest, Tubex, and Typhidot methods.

Groups	Characteristics	TPTest		Tubex		Typhidot	
		Positive	Negative	Positive	Negative	Positive	Negative
Group I	Blood culture positive (n = 28)	28	0	21	7	18	10
Group II	Four fold change of Widal titre (n = 7)	7	0	6	1	5	2
Group III	Widal titre ≥1:320 (n = 13)	9	4	5	8	3	10
Group IV	Blood culture and Widal test negative (n = 44)	24	20	9	35	15	29
Group V	Healthy endemic zone control (n = 20)	0	20	3	17	5	15
Group VI*	Other febrile disease (n = 15)	1	14	1	14	2	13

*Other febrile diseases: visceral leishmaniasis (n = 6), and tuberculosis (n = 9)

To establish upper and lower limits of sensitivity and specificity of the various assays, we used a number of definitions of patient cohorts. If we considered only blood culture-confirmed cases as positive and only patients with other febrile illnesses and healthy controls as negative for enteric fever, the estimated sensitivity and specificity was 100% (95% CI: 87.9%-100%) and 97.1% (95% CI: 85.5%-99.5%) for the TPTest; 75% (95% CI: 56.6%-87.3%) and 88.6% (95% CI: 74.1%-95.5%) for the Tubex assay; and 64.3% (95% CI: 45.8%-79.3%) and 80% (95% CI: 64.1%-90%) for the Typhidot assay, respectively. Alternatively, if we considered all blood culture-confirmed and Widal positive cases as positive, and patients with other febrile illnesses and healthy controls as negative for enteric fever, the sensitivity and specificity of the TPTest was 91.7% (95% CI: 80.5%-96.7%) and 97.1% (95% CI: 85.5%-99.5%); of Tubex, 66.7% (95% CI: 52.5%-78.3%) and 88.6% (95% CI: 74.1%-95.5%); and of Typhidot, 54.2% (95% CI: 40.3%-67.4%) and 80% (95% CI: 64.1%-90%), respectively. If we consider all blood culture-confirmed and Widal positive cases as positive, and patients with other febrile illnesses, healthy controls, and individuals with suspected enteric fever but a negative blood culture and Widal as negative for enteric fever, the sensitivity and specificity of the TPTest became 91.7% (95% CI: 80.5%- 96.7%) and 68.4% (95% CI: 57.5%-77.6%); of Tubex, 66.7% (95% CI: 52.5%-78.3%) and 83.5% (95% CI: 73.9%-90.1%); and of Typhidot, 54.2% (95% CI: 40.3%-67.4%) and 72.2% (95% CI: 61.4%-80.8%), respectively.

### Bayesian latent class modeling

In a latent class model in which sensitivity and specificity of all the diagnostic methods were estimated simultaneously, the sensitivity of TPTest was estimated at 96.0% (95% CI: 87.1%-99.8%), Tubex was 60.2% (95% CI: 49.3%-71.2%), and Typhidot was 59.6% (95% CI: 50.1%-69.3%) (Table 3). Specificity was estimated at 96.6% (90.7%-99.2%) for the TPTest, 89.9% (79.6%-96.8%) for Tubex, and 80.0% (67.7%-89.7%) for Typhidot. The Widal test had a low sensitivity when using a single high titer (14.9%) or four-fold rise in titers (12.6%), but had excellent specificity (86.3% and 100.0%, respectively). Blood culture had intermediate sensitivity at 51.8% (41.2%-62.9%), but 100% specificity.

**Table 3:** Estimated sensitivity and specificity of six diagnostic tests for enteric fever in 92 febrile patients (95% confidence intervals shown in parenthesis), under a Bayesian latent class modeling approach.

Test	Sensitivity	Specificity
**Culture**	51.8% (41.2–62.9%)	100%
**TPTest**	96.0% (87.1%-99.8%)	96.6% (90.7%-99.2%)
**Tubex**	60.2% (49.3%-71.2%)	89.9% (79.6%-96.8%)
**Typhidot**	59.6% (50.1%-69.3%)	80.0% (67.7%-89.7%)
**Widal ≥ 1:320**	14.9% (7.6%-24.7%)	86.3% (75.7%-93.6%)
**Widal 4 fold titer rise**	12.6% (6.2%-22.0%)	100.0% (99.9%-100.0%)

## Discussion

Enteric fever remains an important cause of morbidity and mortality worldwide, especially in infrastructure-limited countries including Bangladesh [2,3]. The clinical diagnosis of enteric fever is difficult because the symptoms and signs of enteric fever are similar to those of many other febrile illnesses [6]. Isolation of *S*. Typhi and *S*. Paratyphi from microbiologic culturing of bone marrow is considered a gold standard for the confirmation of enteric fever. However, the procedure is not clinically practical, especially when considering young children who bear a large component of the enteric fever burden in endemic areas [8]. Microbiologic culturing of blood is thus often used as an alternative diagnostic option when laboratory capacity is available. Unfortunately, the sensitivity of blood culturing is only 40–80%, reflecting in part the low burden of organisms in blood, and often prior use of antimicrobial agents [7]. Results require 2–7 days, but do provide a confirmed diagnosis and an antimicrobial susceptibility profile [20,21]. It is important to note that in our current study, antimicrobial resistance among the *S*. Typhi isolates was very common, with only one oral agent (cefixime) having uniform effective anti-microbial activity. These results underscore the need for an improved diagnostic assay, so that antimicrobial agents that still have activity can be appropriately targeted for use.

The Widal assay has also been available for decades and can be performed using venous blood; unfortunately, the assay has low sensitivity and specificity, especially in endemic zones, and optimally requires comparison of samples drawn at the acute and convalescent stage of illness [22,23]. Nucleic acid amplification assays, such as PCR and LAMP-based assays show promise, but their utility has been hampered in clinical situations by low organisms load and presence of inhibitors in peripheral blood, reagent and equipment expense, and often lack of technical expertise in areas endemic for enteric fever, although such assays may have higher sensitivity than blood culture [7].

As such, to supplement these assays, many clinicians and public health studies rely on other commercially available assays, such as the Tubex and Typhidot immunodiagnostic assays. These assays have the advantage of not requiring extensive laboratory capacity or training, and can be performed on a small volume of venous blood collected at the acute stage of illness. These assays, however, have been limited by less than optimal sensitivity and specificity [19].

It is for this reason that we evaluated the TPTest under field conditions in Bangladesh, comparing results to the other standard used enteric fever diagnostic assays. Our results are quite encouraging. We analyzed performance using two approaches. First, by stratifying the patients by blood culture and Widal reactivity. Second, recognizing the limitations of the absence of a true gold standard, we used a Bayesian latent class modeling approach to estimate sensitivity and performance, comparing across the five imperfect tests.

Using the first approach, the sensitivity and specificity of each test reflects the definition of a true positive or negative used in each analysis. However, in each analysis, the TPTest performed with higher sensitivity and specificity than the Tubex and Typhidot assays. It should also be noted that a number of the patients in our study with suspected enteric fever but who had a negative by blood culture and Widal assay may indeed still have had enteric fever, due to the low sensitivity of these assays. To address this limitation, we analyzed these data using latent class models, a form of statistical analysis that infers an unmeasured, true prevalence, based on the test characteristics and the results of multiple imperfect diagnostics. This approach thereby enables estimation of sensitivity and specificity of diagnostics in the absence of a gold standard. Using such a Bayesian latent class framework thus enabled us to estimate the sensitivity of the TPTest, Tubex, Typhidot and Widal assays among all febrile patients with suspected typhoid fever, rather than restricting our primary analysis to blood culture positivity, which only captures 40–80% of cases [7]. Under this approach, we estimated a very high sensitivity (96%) of the TPTest, in comparison with blood culture, Tubex, Typhidot, and Widal tests [13]. These findings were consistent regardless of model specification. Specificity was high as well, but varied according to prior information about TPTest, Tubex and Typhidot utilized in the model. Specificity estimates for Tubex and Typhidot appeared to be consistent with estimates from a recent meta-analysis, serving as one robustness check [19].

As such, the TPTest appears quite promising. It appears to offer higher sensitivity and specificity than other commonly used assays. At present, the TPTest requires moderate laboratory capacity, results are now available in 24–48 hours, and antimicrobial susceptibility profiles are not provided. However, simplified adaptions are in development, including removal of the requirement of PBMC separation, and CO_2_ during incubation, and removal of the need for an ELISA read out [9–11].

Our study has a number of limitations. It did not include bone marrow aspiration as a gold standard; however, our inclusion of the latent class modeling does allow us to estimate performance using a range of imperfect assays. Our study did not include any patients with confirmed *S*. Paratyphi A bacteremia, so we cannot comment on test performance in such patients in this current study, although we have previously reported that the TPTest detects both *S*. Typhi and *S*. Paratyphi A infected patients [9–11]. Our study is also relatively small, limiting both its context, as well as our ability to assess the impact of age and other factors. It was also of note that in our study, there was no overlap of patients with a positive blood culture and a 4-fold change in Widal titer or high absolute titer. We hypothesize that this may in part reflect the relatively small sample size as well as the deficiencies of the Widal assay in this typhoid-endemic area. In addition, we modeled the diagnostic tests as conditionally independent, but in reality the serologic tests measure immune responses they may be correlated independently from disease. Because of uncertainty in these independent correlations between the multiple serologies, we did not incorporate them in the model, but further characterizing these correlations could improve accuracy of sensitivity and specificity estimates in the future. Despite these various limitations, our study describes a preliminary comparison of the most commonly used diagnostic assays for enteric fever with the evolving TPTest technology, and our results strongly support the continued development of this diagnostic approach.
